# Data-Driven Nonlinear Model Predictive Control for Air Separation Units

**DOI:** 10.1021/acsomega.6c06082

**Published:** 2026-08-13

**Authors:** Valentin Krespach, Nicolas Blum, Martin Pottmann, Shreya Bhatia, Felix Kramer, Lena Tetampel, Parth Shah, Gerhard Zapp, Sebastian Rehfeldt, Harald Klein

**Affiliations:** † TUM School of Engineering and Design, Department of Energy and Process Engineering, Institute of Plant and Process Technology, 9184 Technical University of Munich, Boltzmannstr. 15, 85748 Garching, Germany; ‡ Linde GmbH, 586662 Linde Engineering, Dr.-Carl-von-Linde-Str. 6-14, 82049 Pullach, Germany

## Abstract

In this work, a nonlinear model predictive control framework using a data-driven prediction model is used to control an air separation unit (ASU). The framework is integrated into an industrial automation platform, providing real-time control irrespective of the base layer control system vendor. The approach is a scalable solution that requires minimal effort in model training, leveraging an automated training framework. It is a powerful alternative to physically modeled nonlinear model predictive control (NMPC) and has already been validated by controlling several in-production-critical *Linde* ASUs. Additionally, a multimodel approach is introduced that combines several data-driven prediction models to only exploit the main dependencies of the considered process variables. Furthermore, the approach enables fast implementation at the plant as step tests are not required, which are usually time-consuming and interfere with the normal operation of the plant. Based on two applications, the control performance is presented for different load change scenarios, ensuring compliance with all product specifications. Furthermore, different operating modes, including different products, different equipment, and a different overall control task, can be captured by a single nonlinear model.

## Graphical abstract

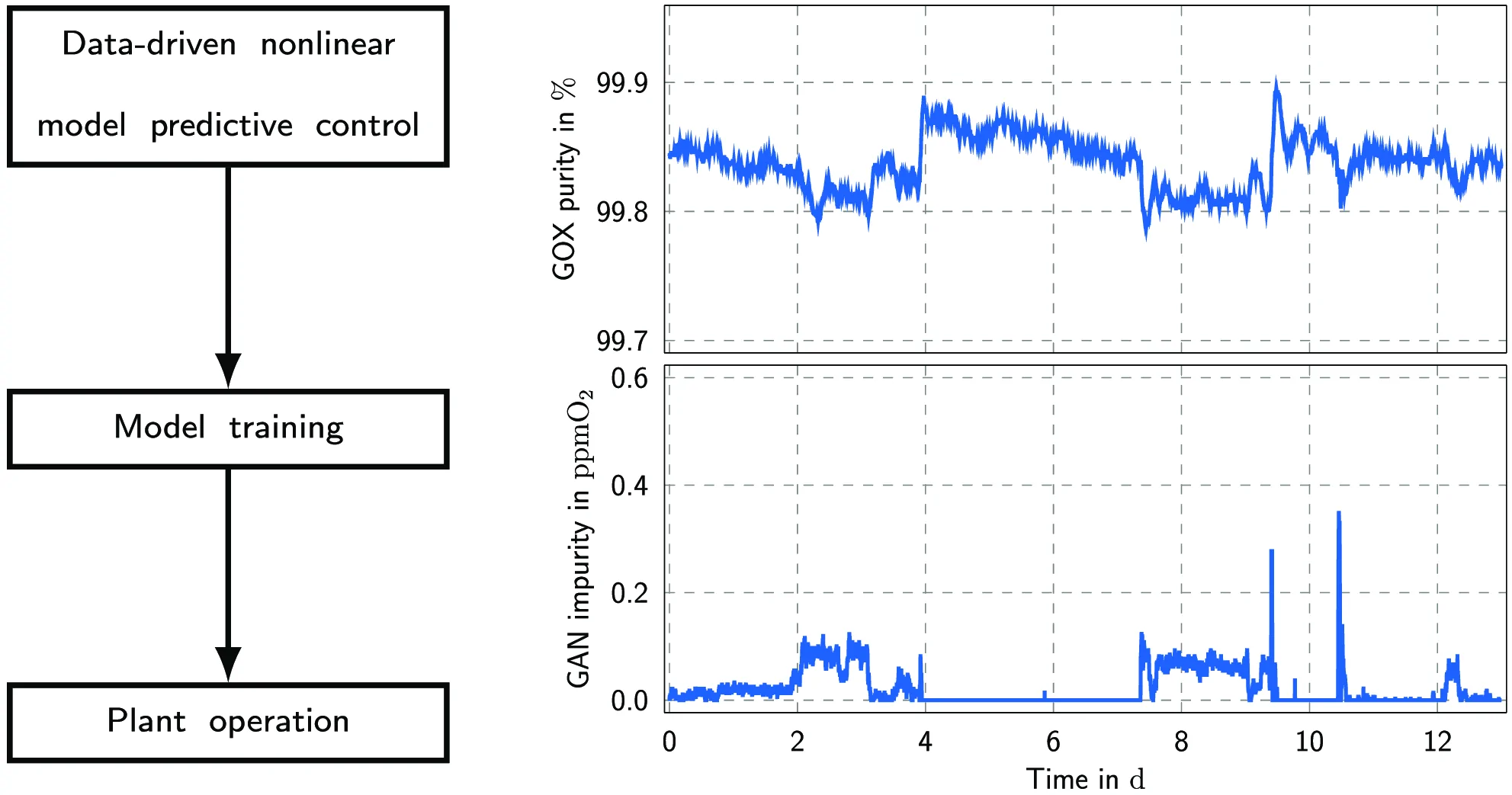

## Introduction

Process control is usually carried out primarily by using basic controllers such as simple proportional–integral (PI) controllers. This feedback-based control strategy ensures continuous set point tracking of the process variables involved. If the control quality needs to be further improved, e.g., by optimizing the overall plant behavior for each sampling step, model predictive control (MPC) concepts are often used. These usually serve as superior control structures and provide suitable set points for the basic lower-level PI controllers.

In addition to the use of linear prediction models, resulting in linear model predictive control (LMPC), a nonlinear prediction model can also be used within an MPC framework. Particularly for highly nonlinear processes, e.g., due to complex thermodynamic relationships, nonlinear model predictive control (NMPC) can achieve significantly higher control quality over the entire operating range. However, the physical modeling of such nonlinear models is often quite challenging, time-consuming, and particularly difficult in terms of ensuring real-time capability.^1^ Therefore, data-driven approaches represent an alternative method for mapping nonlinear relationships.

In this work, an artificial neural network (ANN) is used as a prediction model for control of air separation units (ASU). As first demonstrated by Blum et al.,^2^ this approach requires minimum process knowledge and only historical plant data is used to train the model. While the mentioned paper focused on first tests as a proof of concept under real-world conditions, the approach is in general able to control a multivariable system, which is applied in the present work. Furthermore, the framework is currently used in active control of several in-production-critical *Linde* ASUs. Analogous to the terms LMPC and NMPC, the fully data-driven approach is referred to as AIPC, indicating the usage of artificial intelligence models in the form of ANNs. Additionally to the control of a multivariable system, the present work even further extends AIPC using a multimodel approach. Furthermore, AIPC is suitable to cover completely different operating modes of a plant, which may even have separate control loops.

Other applications of data-driven MPC can also be found in literature. Berberich et al.^3^ proposed a novel data-driven MPC scheme which uses only past measured data for the prediction without any prior system identification step. Furthermore, they analyze the stability and robustness for closed-loop control of linear time-invariant systems. Lee et al.^4^ utilize various machine learning techniques to predict time-series of controlled variables in a petrochemical plant, whereas Zhu et al.^5^ explore a reinforcement learning approach that develops automatic control strategies for a vinyl acetate monomer process plant. Johansen and Foss^6^ introduced a nonlinear modeling framework based on multiple local models that are valid in different operating regimes and interpolated to form a global model in order to enable the representation of nonlinear process behavior over a wide operating range.

In a different study, Wu et al.^7^ proposed a Lyapunov-based MPC approach that employs an ensemble of recurrent neural network (RNN) models to predict nonlinear process dynamics. The RNN models were first trained on extensive open-loop simulation data to accurately capture the system behavior before being integrated into the MPC formulation. Similarly, Bhadriraju et al.^8^ derived multiple local models from historical process data under different operating conditions. These models and their corresponding data sets were subsequently used to train an ANN, which was incorporated into an MPC framework for closed-loop control. Additionally, Son et al.^9^ developed an MPC scheme to improve control of pulping processes affected by feed fluctuations.

A thorough overview of the current state of the art of advanced process control is given in Raven et al.,^10^ which also includes a summary of machine learning-based approaches addressing closed-loop control. Furthermore, comprehensive reviews about MPC strategies using machine learning-based models and time-series ANNs are given in Wu et al.^11^ and Ren et al.^12^

Besides fully data-driven approaches, hybrid models can also be used in plant control combining physical and data-driven modeling. For example, Tsen et al.^13^ used a hybrid approach to develop an MPC scheme for a (semi)­batch polymerization process, whereas Ghosh et al.^14^ use a hybrid model for an LMPC scheme of a crystallization process. Further hybrid approaches in plant control can be found in Bangi and Kwon,^15^ Doyle et al.^16^ and Krespach et al.^17^

In the following, the modeling framework, its specifications and the used setup are presented. Afterward, a multimodel approach is introduced and advantages of data-driven MPC are explained in more detail. Finally, results are shown for two different ASU applications.

## Modeling Framework

Since this work deals with the control of an ASU, its process will be explained below. Subsequently, the fundamentals of MPC are presented and the prediction model used will be described.

### Air Separation

The presented control framework is used in the control of ASUs. The fundamental process is shown in the flowsheet in Figure 1. An ASU separates ambient air into its primary components nitrogen, oxygen, and argon by means of cryogenic distillation. For simplification, air is treated here as a three-component system, and upstream purification steps, such as removal of impurities like water and carbon dioxide, by molecular sieve adsorbers, are not considered.

**Figure 1:**
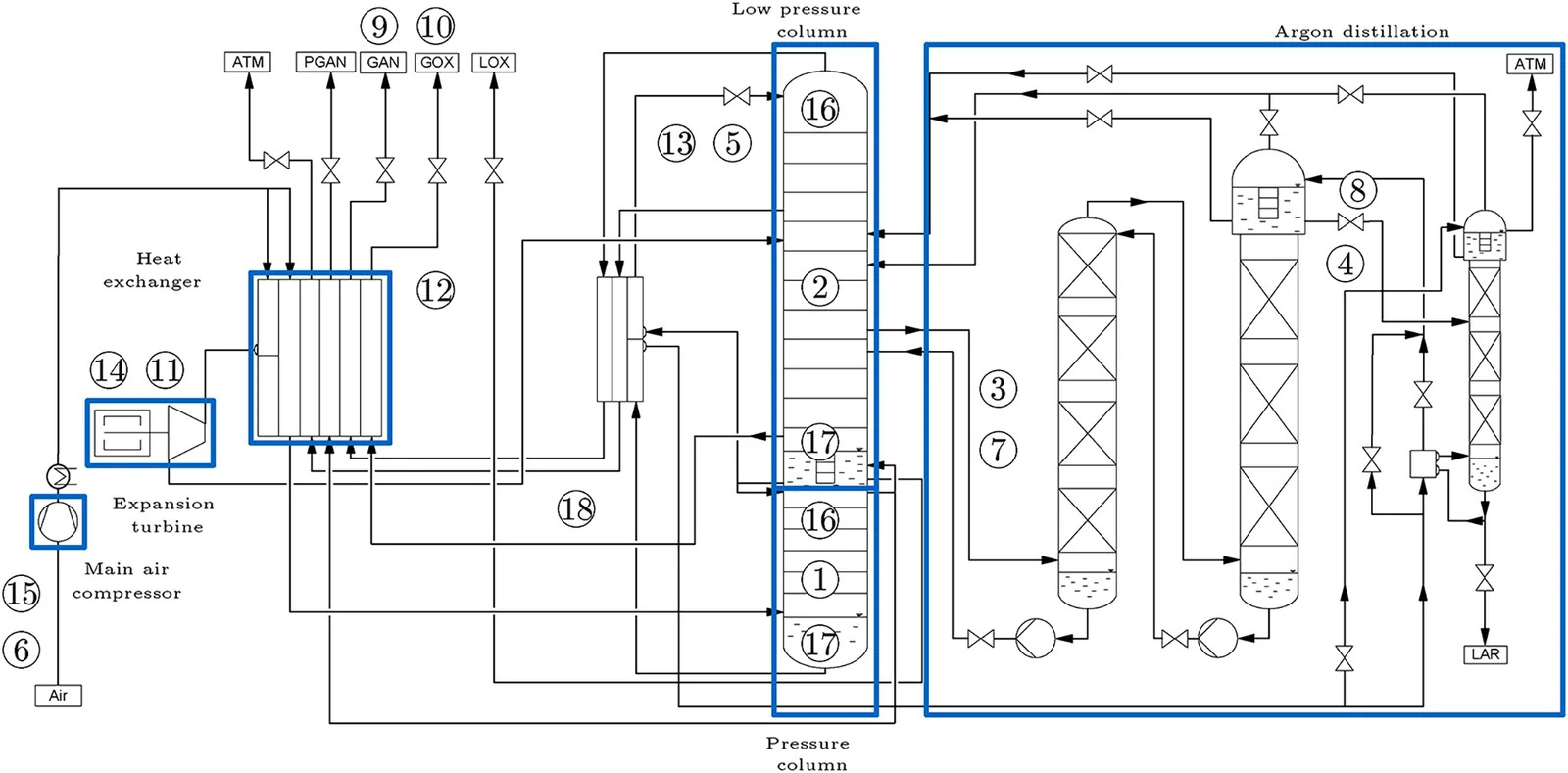
Flowsheet of an air separation unit (ASU). The circled numbers (1) to (18) indicate the position of process variables used as model input and output, see Table 2 and Table 3 in Section ASU1, and Section ASU2. Reproduced from ref 18 under a CC BY license.

In the flowsheet, the air feed is first compressed in the main air compressor, then cooled in a heat exchanger while simultaneously heating the cold product streams from the cryogenic section. One part of the air stream is expanded in a turbine to generate additional refrigeration for the distillation process. Both air streams then enter the so-called *Linde* double column, in which two separate columns are in close thermal contact with each other making them fully heat integrated. Thereby, the lower column (pressure column) is operated at elevated pressure such that the dew temperature at the top of this column exceeds the boiling temperature in the bottom of the upper column, which is operated at a lower pressure (low pressure column).

The pressure column performs an initial separation, while the low pressure column produces oxygen and nitrogen at the desired purities. These products can be withdrawn in pressurized gaseous form (pressurized gaseous nitrogen, PGAN), gaseous form (gaseous nitrogen, GAN; gaseous oxygen, GOX), or liquid form (liquid oxygen, LOX).

In the present flowsheet, also liquid argon (LAR) is produced, requiring additional purification steps (argon distillation). Therefore, an argon-rich stream (argon upflow) is extracted in the lower part of the low pressure column. Since the boiling point of argon lies between those of nitrogen and oxygen, the remaining oxygen is first separated in the raw argon column, implemented here with two columns. The raw argon stream is then separated from nitrogen in the final pure argon column and can be withdrawn as a liquid product.

Depending on the desired amount of liquid product, this loss of refrigeration must be compensated for. In Figure 1, the refrigeration is provided by the expansion turbine, as shown again in Figure 2 (left). However, depending on the plant size and product requirements, alternative ASU process topologies can be implemented. For example, liquid nitrogen (LIN) can then be withdrawn as a product at the top of the low pressure column, or LAR is evaporated in order to produce gaseous argon (GAR). Furthermore, refrigeration can also be provided via the process shown in Figure 2 (right). There, a part of the air stream is further compressed in a booster air compressor (BAC) and, after cooling in the heat exchanger, expanded in a turbine in order to control the refrigeration balance of the plant. Additionally, in this configuration, a third air stream is further boosted in an additional compression stage. After liquefaction in the heat exchanger, this stream is expanded into the pressure column via a throttle valve.^19^ More detailed information on the ASU process, its various configurations and different topologies can be found in Stichlmair et al.,^20^ Moll,^21^ and Hausen and Linde.^22^

**Figure 2:**
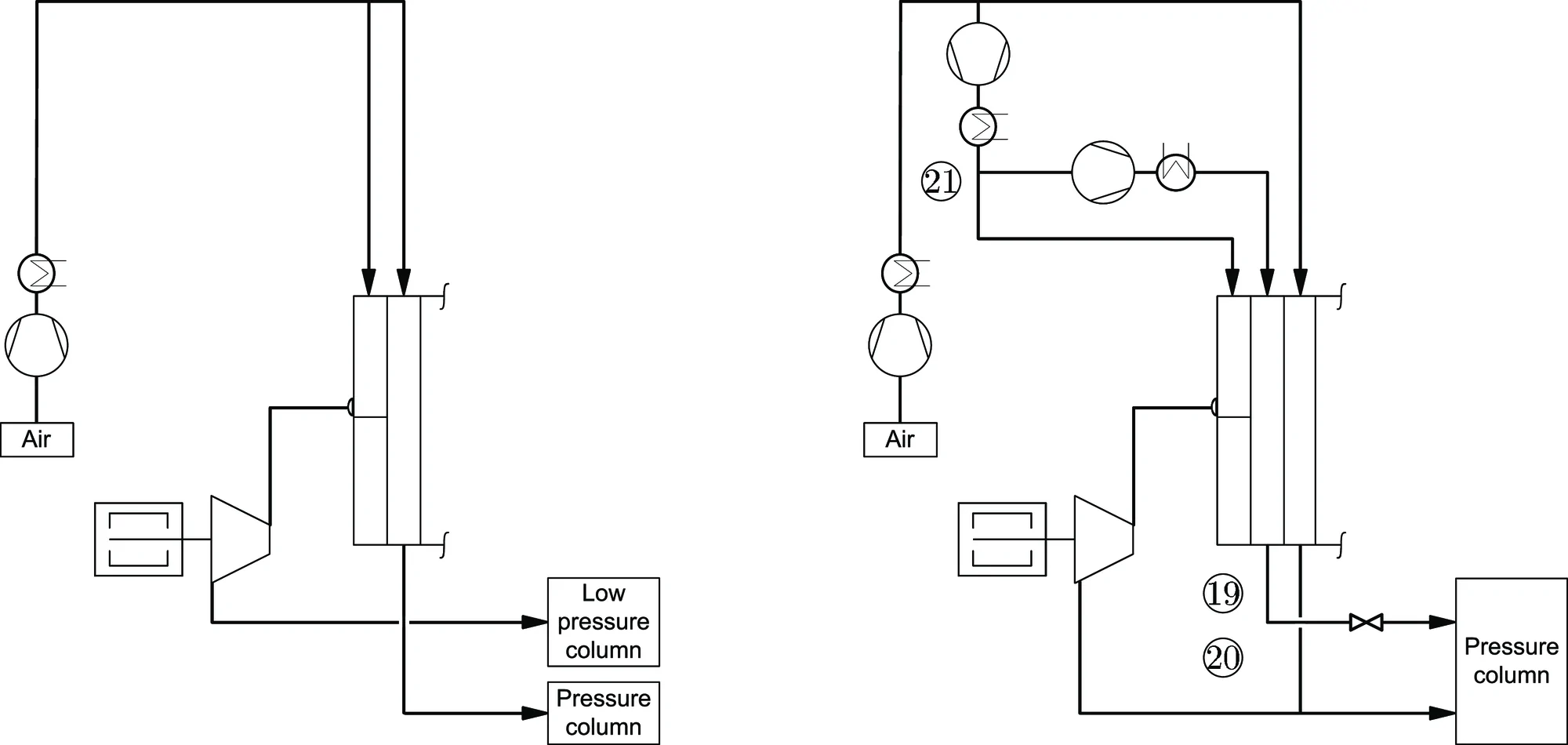
Different possibilities to generate refrigeration for the distillation process. The circled numbers (19) to (21) indicate the position of process variables used as model input and output, see Section ASU2.

### Model Predictive Control

The principle of an MPC is schematically illustrated in Figure 3. First, a discrete-time dynamic prediction model is used to calculate the future behavior of the process. Thereby, it predicts the behavior of the controlled variables (CVs) over a given prediction horizon (here: 90 min). In combination with a desired CV set point trajectory, the prediction is evaluated in the MPC optimizer regarding a previously defined cost function. During the optimization step, the trajectories of the manipulated variables (MVs) are determined (future control action) in order to minimize the cost function. While the MV trajectory is determined for the entire time horizon, only the first step of the MV trajectory is sent to the plant and then the optimization is repeated. For the next time step, the optimization is performed with the then current (measured) state, making it a closed control loop.

**Figure 3:**
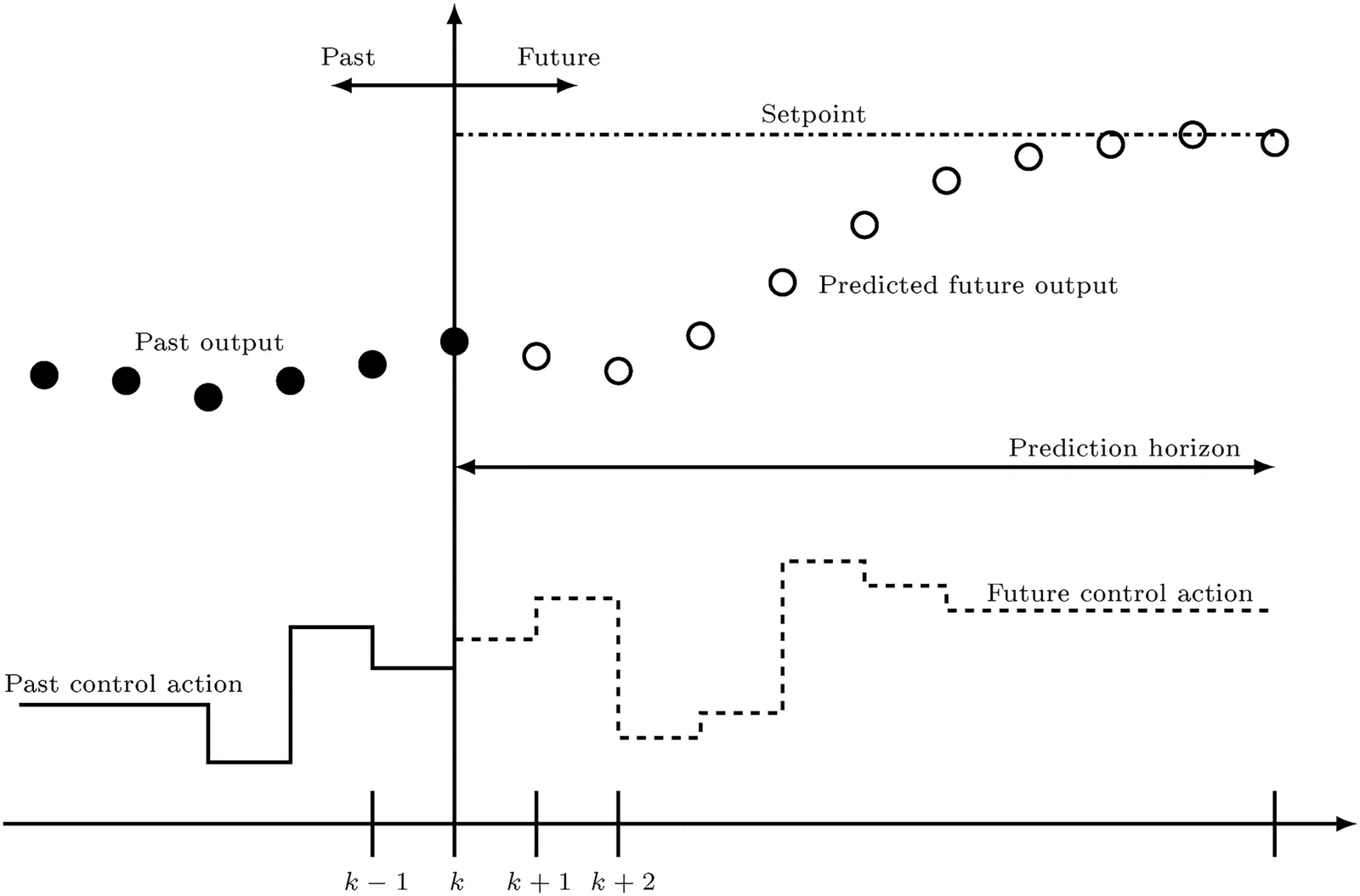
Schematic illustration of the behavior in a model predictive control framework.

For the optimization, a cost function according to eq 1 is used. Thereby, the value of *J* is to be minimized over the prediction horizon containing *K* time steps. In the current work, a gradient descent optimizer with momentum and line search for advanced step size control is deployed for the optimization. Furthermore, the sampling time is set to 30 s. While part of this time is used for safe operation checks, the remainder is used for optimization. Within that time frame, the optimizer performs the maximum possible number of iterations.

$$\min J=\overset{K}{\underset{k=1}{\sum }}\overset{M}{\underset{m=1}{\sum }}\alpha_{m}\Delta u_{m,k}^{2}+\overset{K}{\underset{k=1}{\sum }}\overset{N}{\underset{n=1}{\sum }}\beta_{n}{\left(s_{n,k}-p_{n,k}\right)}^{2}+\overset{K}{\underset{k=1}{\sum }}\overset{L}{\underset{l=1}{\sum }}\gamma_{l}\;f\left(c_{l}-p_{l,k}\right)$$ 1

In the equation of the cost function, α_*m*_ are the weighting coefficients for *M* different MVs used. For each MV, it penalizes large changes in the values between subsequent time steps (Δ*u*_*m*_). The second term reflects the set point tracking errors of the CV values represented by the difference of the set point *s*_*n*_ and the process values *p*_*n*_ for all *N* CV values. Furthermore, state constraints can be considered in the cost function. As a consequence, additional penalties are present only if a current process value (here only used for the CVs) is violating a predefined limit *c*_*l*_. This penalty is defined for *L* constraints and can be expressed by any function *f*, e.g., exponential or softplus. In general, α_*m*_, β_*n*_, and γ_*l*_ represent tuning factors specifying the influence of the respective terms and are determined via case studies.

Depending on the overall objective, additional terms can be incorporated into the cost function, e.g., an additional penalty on the magnitude of a process variable. This is often implemented when considering an economic objective. Examples are given in Caspari et al.^1^ and Schäfer et al.^23^ where they aim to minimize the energy consumption of the ASU process. A general overview on economic model predictive control can be found in Ellis et al.^24^

### Prediction Model

In the present MPC framework, a fully data-driven model is used as the prediction model. This is a fully connected and feedforward ANN consisting of one hidden layer. For the activation function in the hidden layer, the rectified linear unit (ReLU) *f*_ReLU_(*x*) = max (0, *x*) is deployed. As already mentioned, this work is an extension of a previous work by Blum et al.,^2^ which is why only a reference is made here to the publications by Blum et al.^2^ and Krespach et al.^25^ for a more detailed description of the ANN.

Before the ANN can be used for predictions within the MPC framework, a suitable amount of training data is to be collected and sent through preprocessing procedures. This includes applying various filters to remove unsuitable operating conditions such as maintenance, start-up and shutdown of the plant, as well as formatting the data accordingly. Furthermore, the data set is checked for outliers, and an acceptable range is defined for each process variable. If this range is exceeded, the corresponding data point is also removed from the data set. Additionally, the data is standardized (remove mean and scale to unit variance) to ensure a consistent order of magnitude.

During model training in a supervised learning environment, the output of the ANN is evaluated with an available label and the prediction quality is determined using a loss function, which is the mean squared error (MSE) function here. Using the backpropagation algorithm, the gradient of the loss function is propagated backward through the ANN, leading to adjustments of the model weights. For this purpose, the Adam optimization algorithm, as proposed by Kingma and Ba,^26^ is applied. By iteratively optimizing the weights, the model achieves the best possible approximation to the given training data set. Further information on the exact process from data generation and data preparation to model training and evaluation can be found in Krespach et al.^27^

The entire modeling workflow including data preprocessing is set up in a Python environment. For the ANN model training, the machine learning platform Tensorflow^28^ and the ANN library Keras^29^ are used.

## Implementation and Control Task

In general, AIPC offers several advantages of data-driven MPC. One is, the ANN in the prediction model can be trained on any nonlinear relationships, making AIPC a powerful alternative to physically modeled NMPC applications. As a result, nonlinear processes can be handled over a large operating range. In addition, fast implementation without disturbing plant operation can be achieved, as the prediction models are based solely on historical plant data. Compared to conventional MPC solutions, this eliminates the need for time-consuming system identification via step tests, allowing AIPC to be commissioned at the plant in a short amount of time and without interference of the normal plant operation.

Furthermore, AIPC enables for easy adaptation to changing process conditions. These may arise from customer requirements or from the temporal drift of the plant itself. Then, only a retraining is needed, leveraging an automated training framework, to adapt to the changes and AIPC takes over control for the new conditions. Additionally, the maintenance effort is reduced as well.

The following section presents the exact setup used as well as its implementation. It also describes the control task in more detail and lists the model inputs and outputs.

### Setup

For the implemenation of AIPC, *Linde*’s own AOPS application suite, as depicted in Figure 4, is used as an advanced automation platform providing real-time control irrespective of the base layer control system vendor. AOPS allows for the integration of different applications based on a real-time database. In the present setup, AOPS is used for the implementation of AIPC (or MPC in general) and thus takes over the higher-level control tasks. Additionally, a control strategy, here referred to as automatic load change (ALC) system, is implemented in AOPS. Depending on the type of a load change, ALC defines targets for AIPC and, by means of feedforward control, specifies appropriate set points for selected process variables that are required to maintain the desired operating conditions in the new operating point. These targets and set points are derived from process knowledge and are expressed in the form of a mathematical model. Further on, within AOPS an automatic sequence transition (AST) system is implemented to support start-up and shutdown procedures.

**Figure 4:**
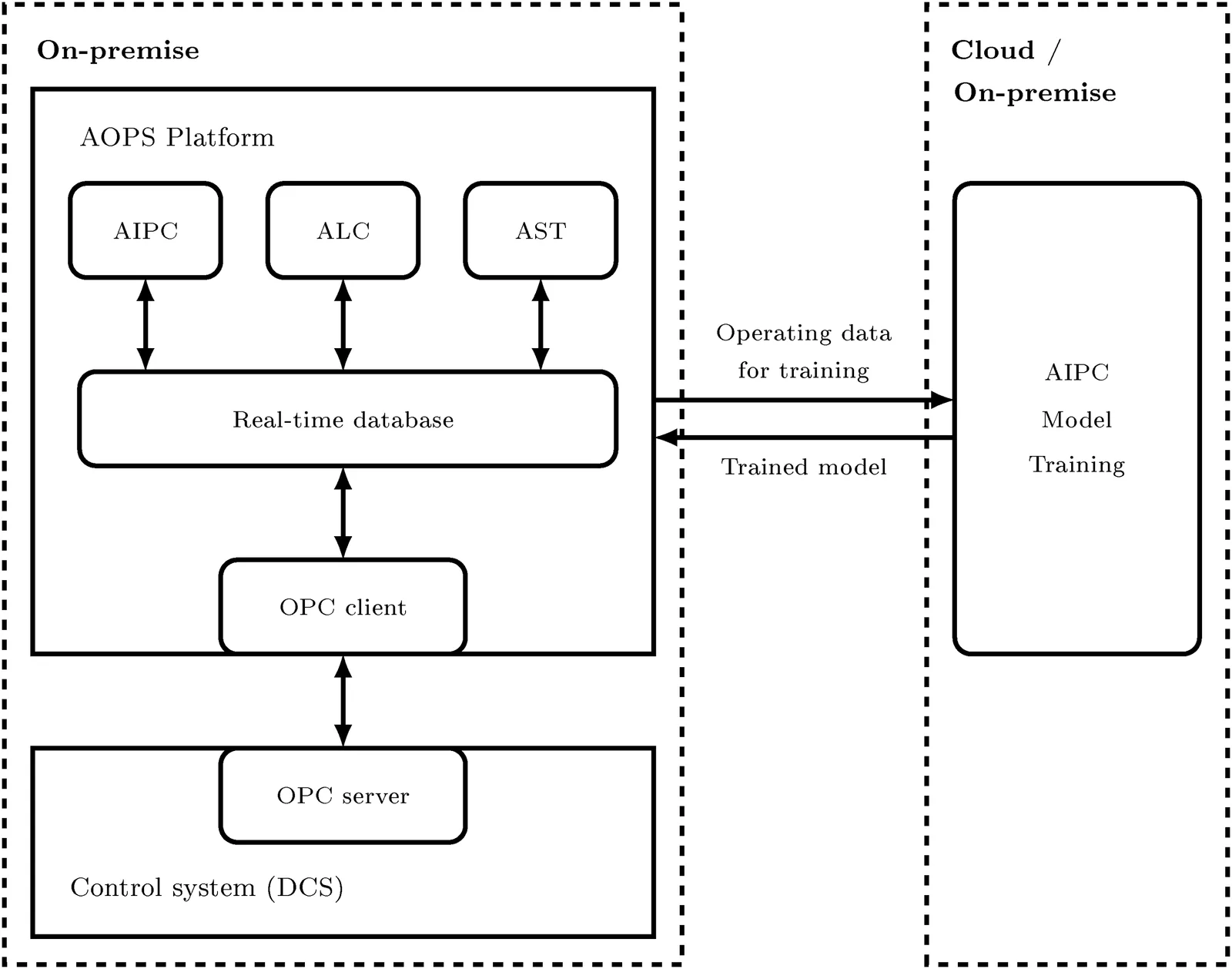
Illustration of the used setup.

In general, AOPS is able to communicate with the distributed control system (DCS) of the plant via OPC. Furthermore, AOPS provides operating data for training the data-driven prediction model, which can be used for plant control, afterward.

In the following sections of this work, results obtained from the application of AIPC will be presented for both a real-world plant as well as a digital twin (DT). For this purpose, a virtual environment is implemented that replicates the setup at a real plant as accurately as possible and can be used in order to avoid disrupting plant operations during the controller development and to allow freedom for testing. The previously mentioned AOPS application suite is implemented in the virtual environment just as it is in the corresponding real plant. However, the OPC client is directly connected to the DT. In this work, the DT as a substitute for the real-world plant is implemented in the simulation software UniSim Design.

In general, a DT is a highly detailed dynamic plant model, and can be used as a virtual representation of a real plant. As it is modeled with high fidelity, it can adequately replace the real plant as a controlled system.^30^ Therefore, a DT can also be used in order to develop new control strategies before implementing them in the plant.^18,31,32^ Furthermore, the DT can be used for data generation. It is noted that the base controllers, e.g., level controllers, are already included as a part of the DT.

### Control Task

As previously mentioned, the current work examines two different applications, which will be referred to as ASU1 and ASU2 in the following, resulting in multivariable control problems of different size. The primary objectives of the control system are to provide the required amounts of products while maintaining purity specifications of the nitrogen, oxygen and argon products in both gaseous and liquid form. In this work, the product specifications are set to the values shown in Table 1. It is pointed out that the product specifications for nitrogen and argon are expressed with their remaining oxygen amount, therefore later referred to as impurities. The two values for the constraint limits are also shown. Thus, if the process value exceeds these constraint limits, an additional penalty in the MPC cost function is activated. In the current work, the constraints are implemented as soft constraints and help ensure compliance with strict purity specifications. This means that if a constraint limit is exceeded, the optimization procedure focuses more on these process variables, thereby ensuring that corrective action is taken early on, before the actual purity limits are violated.

**Table 1:** Product Specifications and Constraint Limits for the Considered Products

product	constraint limit	product specification
nitrogen	0.8 ppmO_2_	1.0 ppmO_2_
oxygen	99.58%	99.50%
argon		1.0 ppmO_2_

Regarding the inputs of the prediction model, the ANN used here is built according to the structure of a nonlinear autoregressive model with exogenous inputs (NARX). As a consequence, preceding CV and MV values (here: 20 for each process variable) are used as model inputs. In general, the selection of CVs and MVs corresponds to the respective higher-level control loops of a plant. They influence the required product specifications and pose significant interactions with each other making the use of a multivariable controller advantageous. Further model inputs are the constraint variables as well as certain additional sensor variables, which are used to model the system behavior more realistically and accurately. The number of model outputs is determined according to the number of CVs and constraints. It is worth mentioning that, in addition to the control loops covered by AIPC, there are also lower-level PI controllers present, such as those used to control liquid levels, flow rates or pressures.

#### ASU1

The control task in application ASU1 considers the ASU plant shown in Figure 1, mainly producing GAN, GOX and LAR. However, a DT of this plant is used in order to avoid disrupting plant operations and to allow freedom for further AIPC developments. Therefore, the virtual environment described in Section Setup is deployed here. The control task includes a multivariable system consisting of four control loops. Accordingly, four CVs and MVs are listed in Table 2. Each process value is marked with a corresponding number indicating its position in the flowsheet in Figure 1.

**Table 2:** List of Controlled Variables (CVs) and Manipulated Variables (MVs) for Application ASU1^a^

controlled variables (CVs)	manipulated variables (MVs)
• midpoint temperature in pressure column (①)	• valve position for reflux of low pressure column (⑤)
• midpoint temperature in low pressure column (②)	• air feed flow rate (⑥)
• oxygen concentration in argon upflow (③)	• argon upflow (⑦)
• raw argon impurity (④)	• raw argon flow rate (⑧)

a The corresponding numbers indicate the position of the process variable in the flowsheet in Figure 1

While the process values of the CVs are to be controlled to their respective set points, the product specifications for the two main products GAN and GOX need to be ensured. They are therefore considered as constraints (listed in Table 3) in the MPC cost function (eq 1). For the argon product, no constraint limit is used. Here, only the raw argon control loop (fourth CV and MV in Table 2) is responsible for achieving its purity. Table 3 also lists certain additional sensor variables, which are used as model inputs.

**Table 3:** List of Constraint Variables and Additional Sensor Variables for Application ASU1^a^

constraint variables	sensor variables
• GAN impurity (9)	• turbine inlet flow rate (11)
• GOX purity (10)	• GOX product flow rate (set point and process value) (12)
	• temperature at reflux valve for low pressure column (13)
	• turbine inlet temperature (14)
	• air feed pressure (15)
	• column pressures (16)
	• liquid level in pressure and low pressure column sumps (17)

a The corresponding numbers indicate the position of the process variable in the flowsheet in Figure 1.

The data required for model training is generated using the virtual environment and consists of both steady-state and dynamic data. Therefore, various load change scenarios were simulated, and different operating points within the considered operating range were reached.

#### ASU2

Application ASU2 deals with an in-production-critical *Linde* ASU. This plant primarily differs from the previous application ASU1 in generating refrigeration for the distillation process. For this purpose, the process shown in Figure 2 (right) is used, with additional compressors (BAC) and a throttle valve installed. Furthermore, it is possible to produce all three air components in both liquid and gaseous form. Therefore, depending on the desired amount of liquid product, more or less refrigeration is generated.

In general, the control task is similar to that in application ASU1, however, now 5 CVs and 6 MVs are considered. As listed in Table 4, the first three CVs are similar to application ASU1, with the difference that the two temperature measurements in both the pressure and the low pressure column are measurements of the oxygen concentration, now. Furthermore, the oxygen concentration in a nitrogen-rich side stream of the low pressure column is used as a CV. This stream is primarily used for regenerating the adsorbers during air purification but also contributes to an increase in nitrogen purity at the top of the low pressure column. Additionally, the temperature upstream of the throttle valve is used as a CV.

**Table 4:** List of Controlled Variables (CVs) and Manipulated Variables (MVs) for Application ASU2^a^

controlled variables (CVs)	manipulated variables (MVs)
• oxygen concentration in pressure column	• valve position for reflux of low pressure column
• oxygen concentration in low pressure column	• air feed flow rate
• oxygen concentration in argon upflow	• argon upflow
• oxygen concentration in nitrogen-rich stream (18)	• reflux of pressure column
• temperature throttle valve (19)	• GOX product flow rate
	• flow rate throttle valve (20)

a The corresponding numbers indicate the position of the process variable in the flowsheets in Figures 1 and 2.

For the MVs, the first three process variables are identical to application ASU1. Additionally, the reflux into the pressure column, the GOX product flow rate, and the flow rate through the throttle valve are used. It is worth mentioning that in this application, the argon product is controlled by PI controllers and not via AIPC, as is the case with the fourth control loop in application ASU1.

The constraints here correspond to those in application ASU1. Also, the additional sensor variables used mostly correspond to those shown in Table 3 and relate to liquid levels, flow rates, and pressures. In addition, the BAC flow rate (21) in Figure 2) is also used here.

A distinctive feature of the considered ASU is that it can be operated in two quite distinct operating modes, each yielding different products. In the gas mode, only gaseous products are produced, whereas in the liquid mode, liquid products are the main output in addition to some production of gaseous products. Since these liquid flows are not available in the heat exchanger to cool down incoming air feed flows, refrigeration is lost in the process, which must be compensated for by the refrigeration system. This means that the described plant configuration applies only to the liquid mode. In contrast, less refrigeration is required in the gas mode, which is why no additional compression in the BAC takes place, here. This leads to the fact that the throttle valve control loop, consisting of the upstream temperature as CV and the flow rate as MV, is not active in the gas mode. Consequently, the liquid mode and the gas mode reflect significantly different operating modes including different products, different equipment, and a different overall control task, which is also the reason why the use of an LMPC with a single control matrix and/or controller configuration is not suitable in this plant.

The data required for model training is derived from historical data from the plant itself and consists of various operating points, load change scenarios, and different operating modes of the plant.

Since ASU2 is an in-production-critical ASU and therefore represents a higly safety-critical industrial process, it is worth mentioning that automated fallback solutions are in place to ensure the closed-loop stability of the plant. For example, if the prediction quality of the model decreases, if purity specifications are no longer met, or if problems arise in the IT infrastructure, the system will automatically switch to the base-layer control system consisting of PI controllers.

## Multimodel Approach

In general, the current AIPC setup is being used for the control of several in-production-critical *Linde* ASUs, demonstrating its robustness for industrial reliability and implementation maturity. As explained, this setup is also used in the current work. In application ASU1, a control task consisting of four control loops is faced, ensuring multivariable control of all three ASU products. In order to even further improve AIPC performance here, a multimodel extension is introduced combining several prediction models. It is pointed out that the multimodel approach is only applied to application ASU1 and not to ASU2.

Each time a new prediction model is trained, it should be assessed using a number of evaluation methods before it is used to control a plant. This helps to avoid costly interruptions or poor performance. This involves, e.g., examining the value of the loss function, which is supposed to converge to the lowest possible value during model training. Furthermore, multiple prediction models are trained simultaneously during each training session so that the model with the best performance can be selected. Further on, a common model evaluation tool is the use of step responses, in which the set point of one MV is changed instantaneously to observe the process response and to understand the behavior of the system. Analogous to linear MPC, this results in a control matrix that is exemplarily illustrated in Figure 5. It includes step responses recorded for each CV/MV pair.

**Figure 5:**
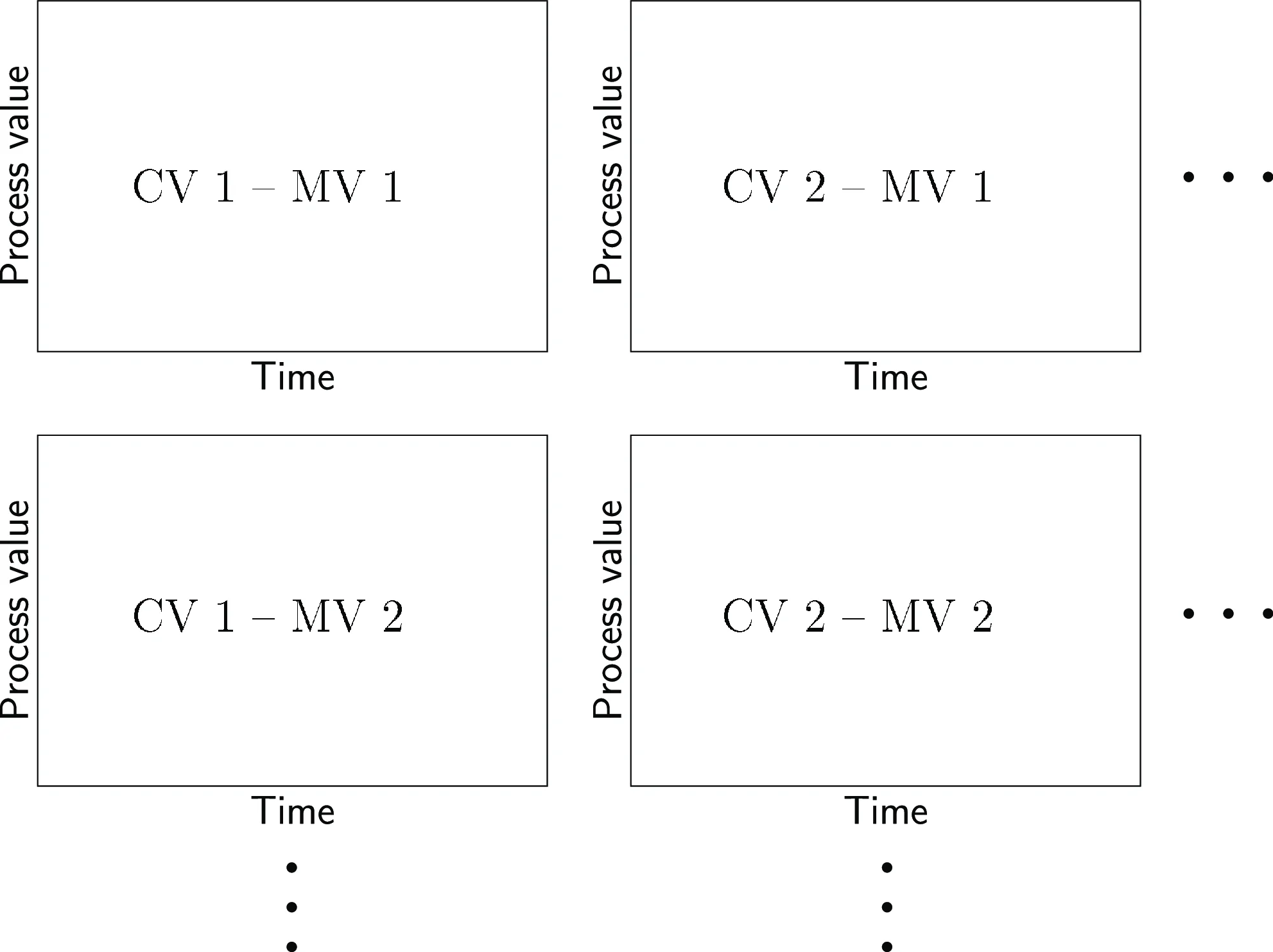
Control matrix showing step responses for all CV/MV pairs.

In common LMPC applications, only individual elements of the control matrix are filled solely showing the most important dependencies. Consequently, if a process possesses a large number of CVs and MVs and is therefore highly complex, only the most important relationships are considered, which sometimes results in a very sparsely occupied control matrix. This also means that minor dependencies or conflicting relationships between different process variables are neglected. In addition, parts of a plant are treated independently and only the associated process variables are considered.

Since for nonlinear models such as ANNs the superposition principle does not hold, AIPC models are based on the assumption that all input variables can affect all output variables. If an input variable has little or no impact on an output variable, then the corresponding relationship found by the data-based model is often also of negligible magnitude compared to more important input variables. Therefore, the multimodel approach, introduced in this section and schematically illustrated in Figure 6, can be seen as the nonlinear extension of setting up a sparse control matrix or *variable blocking* in the linear case. Specifically, input and output variables of a particular process area can be grouped together into one variable block, while variables associated with another process area are grouped into another block. This could go as far as assigning a different input variable set for every process output, implying that different submodels are determined for every process output.

**Figure 6:**
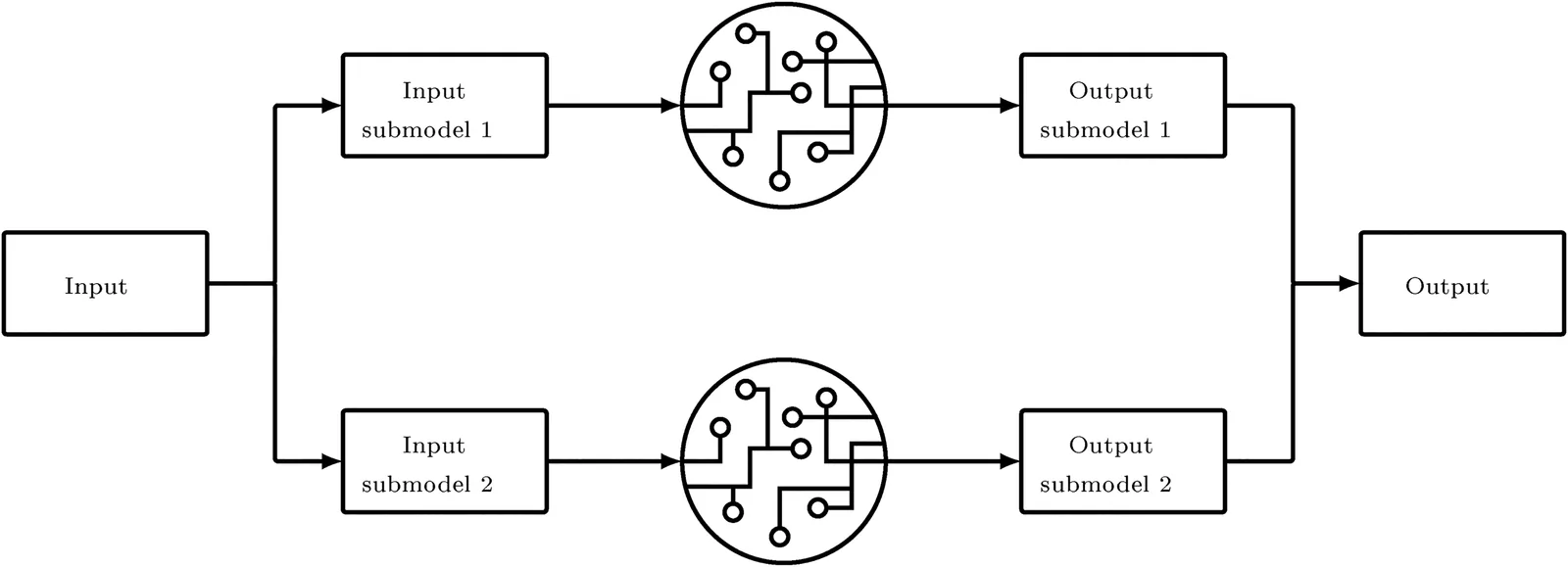
Illustration of the multimodel approach introduced to AIPC.

The main idea behind the multimodel approach is to connect several individual prediction models in a parallel way. Each submodel is trained separately from each other using input values from the same set of process variables. Subsequently, the model outputs of the different submodels are combined into one overall prediction model. Thus, the subsequent MPC optimizer recognizes only one (overall) prediction model, no matter how many submodels are used for the model predictions.

In the current work, and for application ASU1 only, two submodels are deployed. Thereby, one submodel represents the first three control loops listed in Table 2. Consequently, the second submodel solely represents the raw argon control loop influencing the final argon product. By doing this, it exactly applies the feature of separating relatively independent parts of the plant. The three control loops combined in the first submodel relate to process variables that significantly influence each other. Thus, the major advantage of MPC in general can be exploited here and an enhanced multivariable control system can be established. In contrast, the process variables of the raw argon control loop influence a different process area and can therefore be considered on their own.

More specifically regarding the control of the argon loop, it is noted that using a multimodel configuration comprising two submodels performs better than a single-model configuration. The reason for this is that in a single-model configuration all elements in the control matrix are utilized, which introduces unnecessary dependencies into the prediction model. In the present use case, this leads to a reduction in the prediction quality and overall performance. As for LMPC applications, it is therefore advisable to eliminate these dependencies with the introduced multimodel approach. This results in a sparse control matrix exploiting only the most important dependencies.

Another advantage of the multimodel approach is the possibility of handling control loops with major differences in their settling times. In fact, the argon distillation part of an ASU is a system showing slow behavior. Therefore, it turns out beneficial to consider the argon control loop in a separate prediction model.

## Results and Discussion

In the following, the results are presented for both application ASU1 and application ASU2.

### ASU1

In order to evaluate the performance of the presented AIPC control framework regarding application ASU1, a load change from 100% plant load to a lower plant load is always performed in the following. Then, after a certain time, the plant load is changed back to the initial operating point. The plant load is defined by its GOX demand, which is set by a directly connected customer. Therefore, only the GOX product flow rate needs to be varied in order to perform load changes here. All other process variables are then adapted via the implemented control system. As already mentioned, the control system also includes the ALC system. In order to reach the desired operating point more efficiently, the set points of the temperatures in both pressure and low pressure column are adapted as well as the liquid levels in the column sumps, here. The rate of a load change for moving between two operating points is set to 0.5% in plant load per minute, which also applies to the corresponding real-world ASU.

The results of a 20% load change are shown in Figure 7. The corresponding GOX profile is depicted in the upper left plot. It can be recognized that the process value (dashed line) requires 40 min to reach the desired set point (solid line) after each load change. It is noted that a load change of 20% is a typical scenario which also occurs in the corresponding real-world ASU.

**Figure 7:**
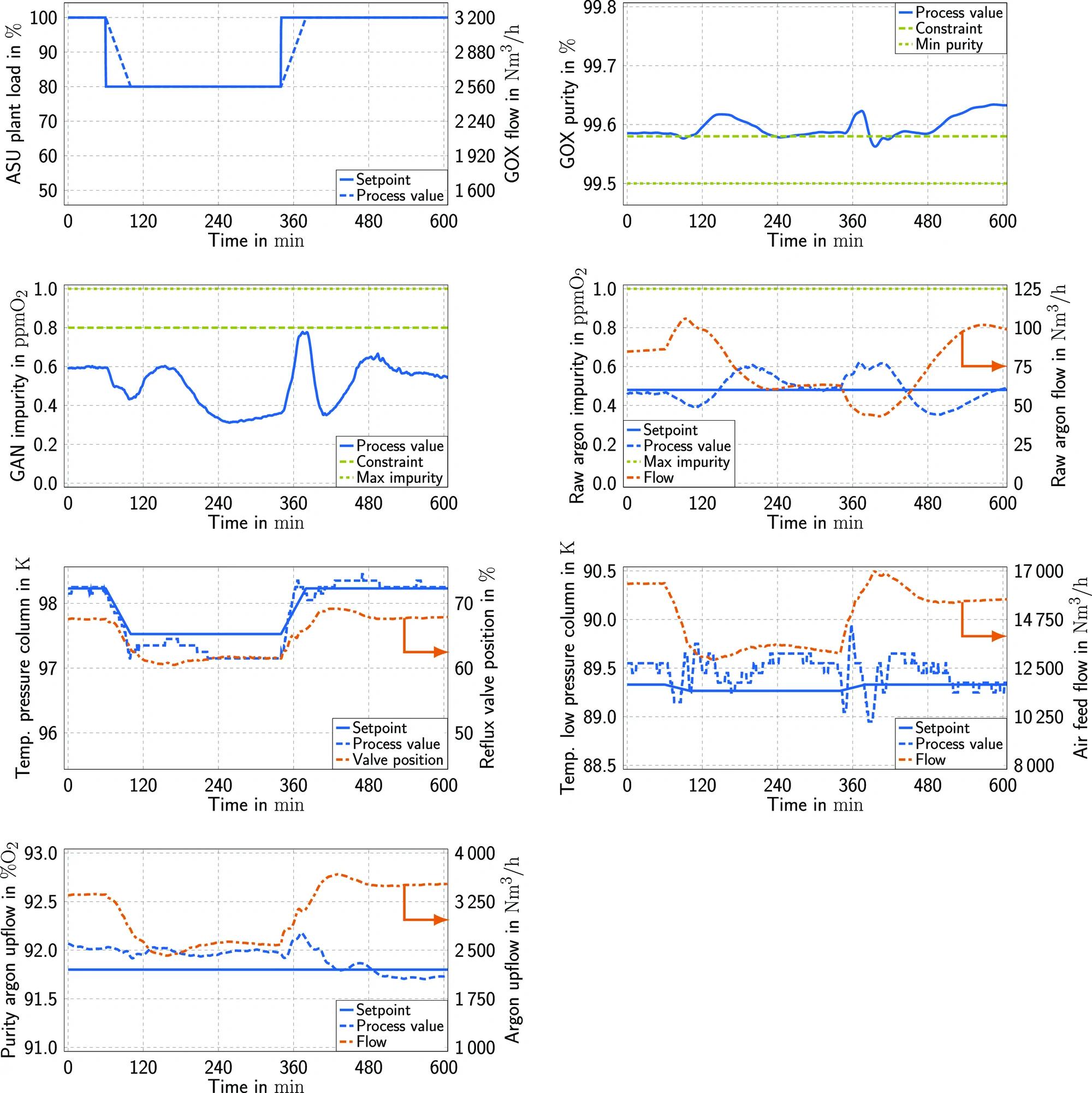
AIPC performance for a 20% load change.

The most important criterion for a production plant is compliance with its product specifications. Therefore, the product impurity and product purity for GAN and GOX, respectively, are shown in the upper middle left and upper right plots. The associated constraint limits (dashed line) and product specifications (dotted line) from Table 1 are also plotted, respectively. It is clearly visible that both product specifications are successfully maintained throughout the entire time period. For the GOX product, the set constraint limit is exceeded, though, only at very few points in time. This is not critical for plant operation, it merely activates an additional term (third term in eq 1) in the cost function of the MPC optimizer.

The remaining four plots show the courses of the four CVs (sepoint: solid lines, process value: dashed lines) and MVs (dashdotted lines) used. Although MPC in general is a multivariable problem in which an MV cannot necessarily be completely assigned to a certain CV, in this case the selected CV/MV pairs in the respective plots represent the principal correlations. Thereby, the product purity for LAR is implicitly shown by the raw argon impurity in the upper middle right plot. Here again, it can be recognized that the product specification (dotted line) is successfully maintained throughout the entire time period.

The two lower middle plots show the midpoint temperatures of the pressure column and low pressure column. The set points are adjusted depending on the load change via the implemented ALC system. Furthermore, the two MV courses for the reflux valve position and air feed flow rate show load-dependent behaviors.

In the lower left plot, the purity (or oxygen concentration) in the argon upflow is shown. The process value is kept close to the constant set point over the considered time period. Major changes only occur during the two performed load changes. In addition, the flow rate of the argon upflow is shown. It again behaves similarly to the specified plant load.

The course of the raw argon flow rate (upper middle right plot) also indicates a slightly load-dependent behavior, although this is not quite as pronounced as in the other three MVs. On the one hand, the large settling time of the control loop results in a slow behavior of the process variables, so interferences in the process affect the process variables with a time delay. On the other hand, though, the behavior is also heavily related to changes in the column holdups and liquid levels due to the performed load changes.

In summary, the individual set points of the CVs are well maintained, thus guaranteeing successful load changes while complying with the required product specifications.

While the load change of 20% plant load described above reflects a realistic scenario of the ASU, a second scenario is focused on below. This is intended to represent a less frequent scenario comprising a load change of 50%. Accordingly, the plant load is changed between 100% to 50%, indicated by the GOX demand in the upper left plot of Figure 8.

**Figure 8:**
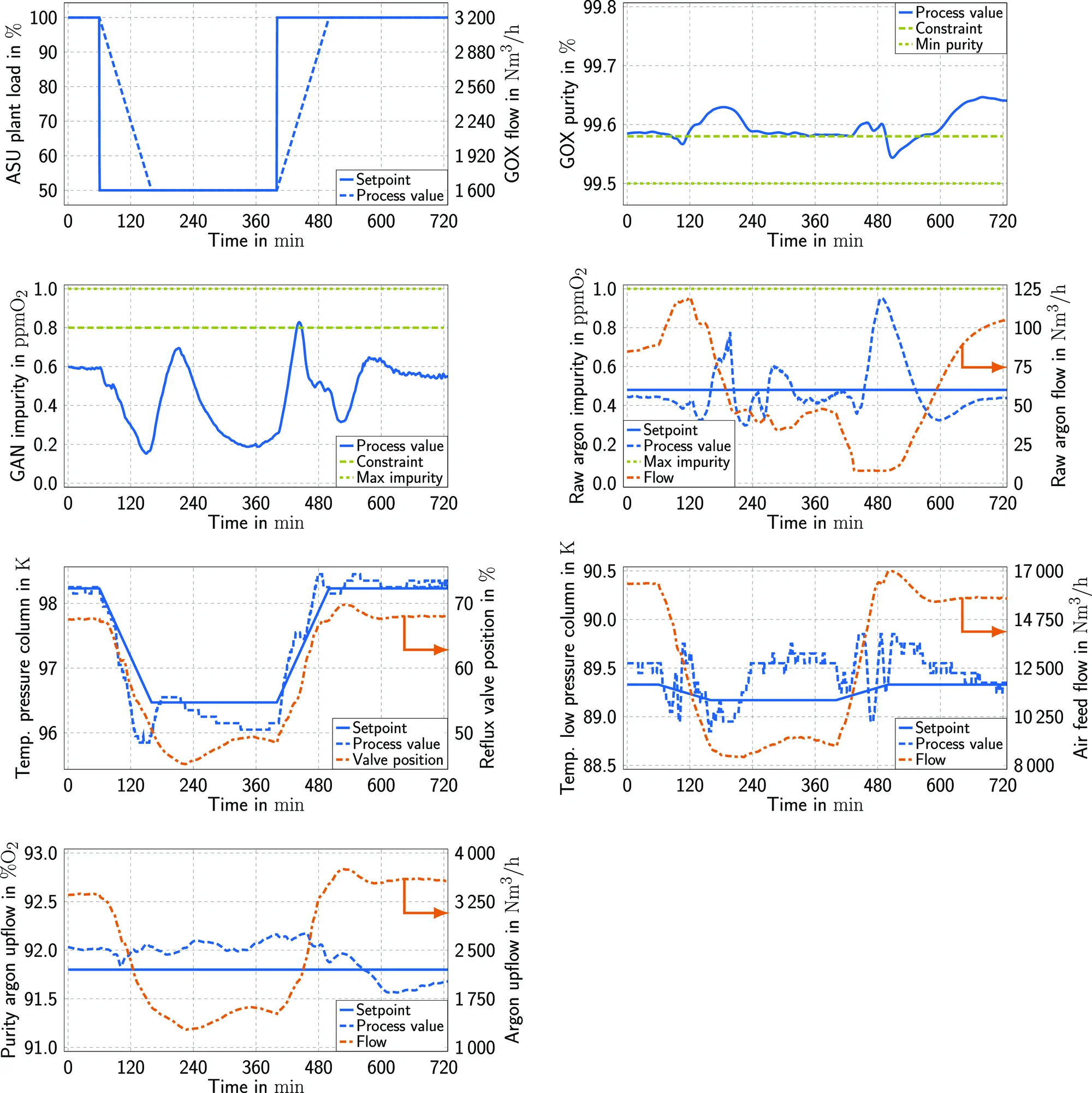
AIPC performance for a 50% load change.

It is clearly visible that the product specifications for GAN, GOX, and LAR are maintained again throughout the entire time period. Overall, there are now larger changes in the time courses of all three products. This can be attributed to the extensive load change, which represents a strong interference in plant operation and is rather unlikely to occur in real-world ASUs. Nevertheless, this scenario shows that AIPC can also handle such large load changes.

Considering the different MVs in the remaining plots, they again show a load-dependent behavior, though, now to an even greater extent than in the previous scenario. It ensures that the CV set points are reasonably maintained in order to ultimately guarantee the required product specifications.

### ASU2

For the evaluation of the control performance of AIPC in application ASU2, a 13-day period is considered below. The plots to follow show the time histories of process variables that were observed in the plant.

The BAC flow can be seen as an indicator of the operating mode of the plant as it is primarily used to provide refrigeration for the distillation process. Therefore, its trend is shown in the upper plot in Figure 9. It is visible that the BAC flow exhibits a flow of 0 over several days, thus illustrating the gas mode of the plant. Additionally, the flow and pressure of the throttle valve downstream also show a value of 0 (middle plot). In contrast, the process variables indicate a positive value during the remaining periods illustrating the liquid mode of the plant. Thus, the plots clearly show that the two operating modes differ fundamentally in terms of the equipment used.

**Figure 9:**
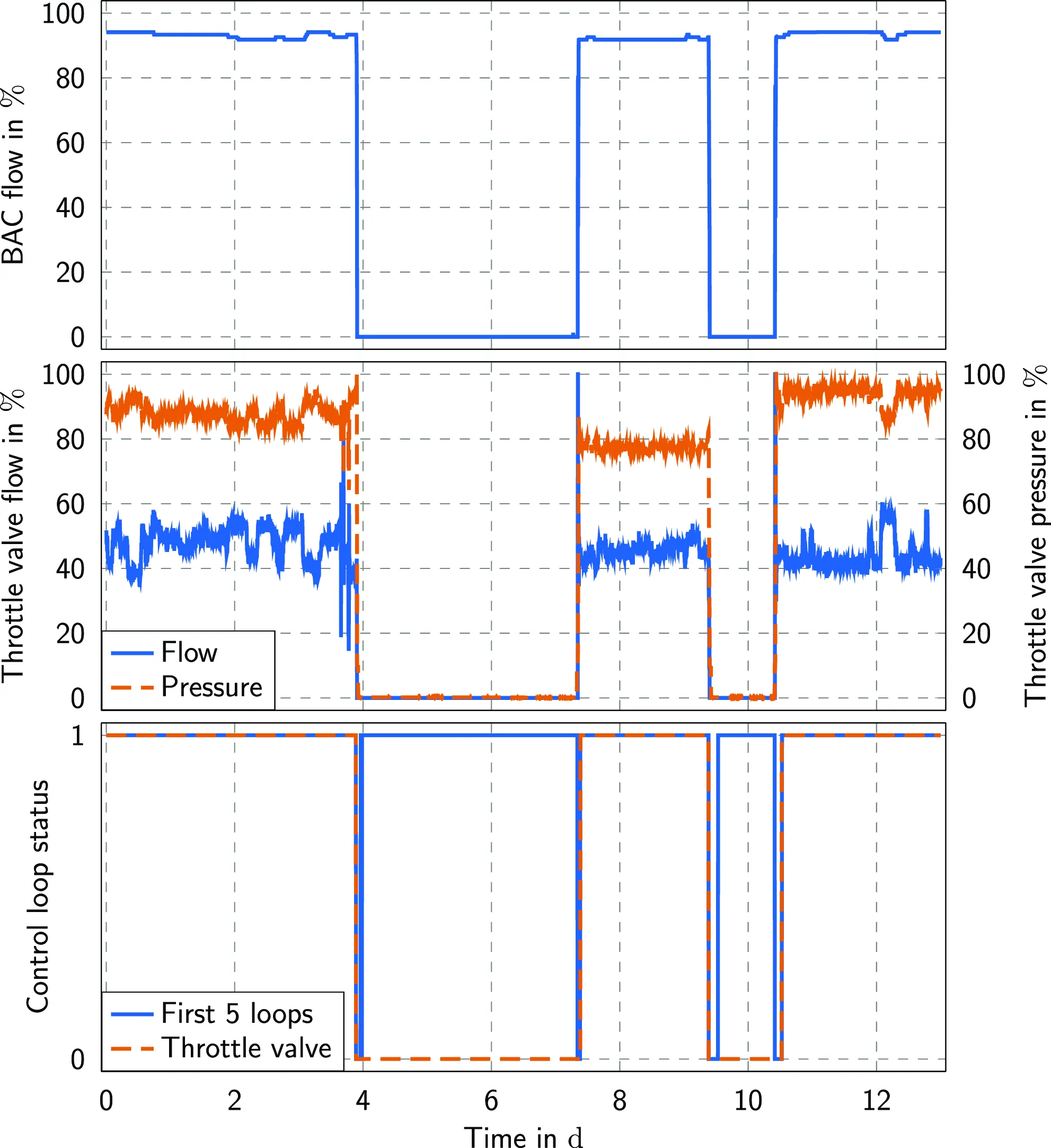
Plant data for the BAC flow, throttle valve and control loops used.

It is important to mention that both operating modes are captured by a single nonlinear model. Depending on the operating mode, different *regions* of the model will be in use without the need for changing the input/output variable sets. Consequently, no switching between control models is required here, which would be necessary when using an LMPC solution.

Figure 9 (lower plot) also shows the status of the control loops used by AIPC. In the liquid mode, all 6 MVs are active and are used by AIPC to maintain the set points of the CVs and to guarantee the required product specifications. In contrast, in the gas mode, it is evident that the throttle valve control loop is not being used. Since the throttle valve does not contribute to the required refrigeration of the plant in the gas mode and there is no flow through this part of the plant, the control loop is not active here, thereby illustrating the different control tasks for AIPC in the two operating modes.

Furthermore, it can be seen that, when switching between the individual operating modes, all control loops are temporarily deactivated. This is due to the fact that AIPC as a control framework is not capable of turning individual machines and equipment on and off, so this process is currently performed manually by the operator at the considered plant. Alternatively, the AST described in Section Setup can also be used for this purpose which could be part of future development. Once this is completed, however, AIPC is reactivated for plant control.

Even within these operating modes, the plant still needs to meet changing customer requirements. Therefore, the plant is operated at different operating points and load change scenarios are performed. As these are primarily characterized by the GOX product flow and the air feed flow in the considered plant, both process values are shown in Figure 10. It can be seen that numerous load change scenarios are performed in both operating modes of the plant during the observed time period.

**Figure 10:**
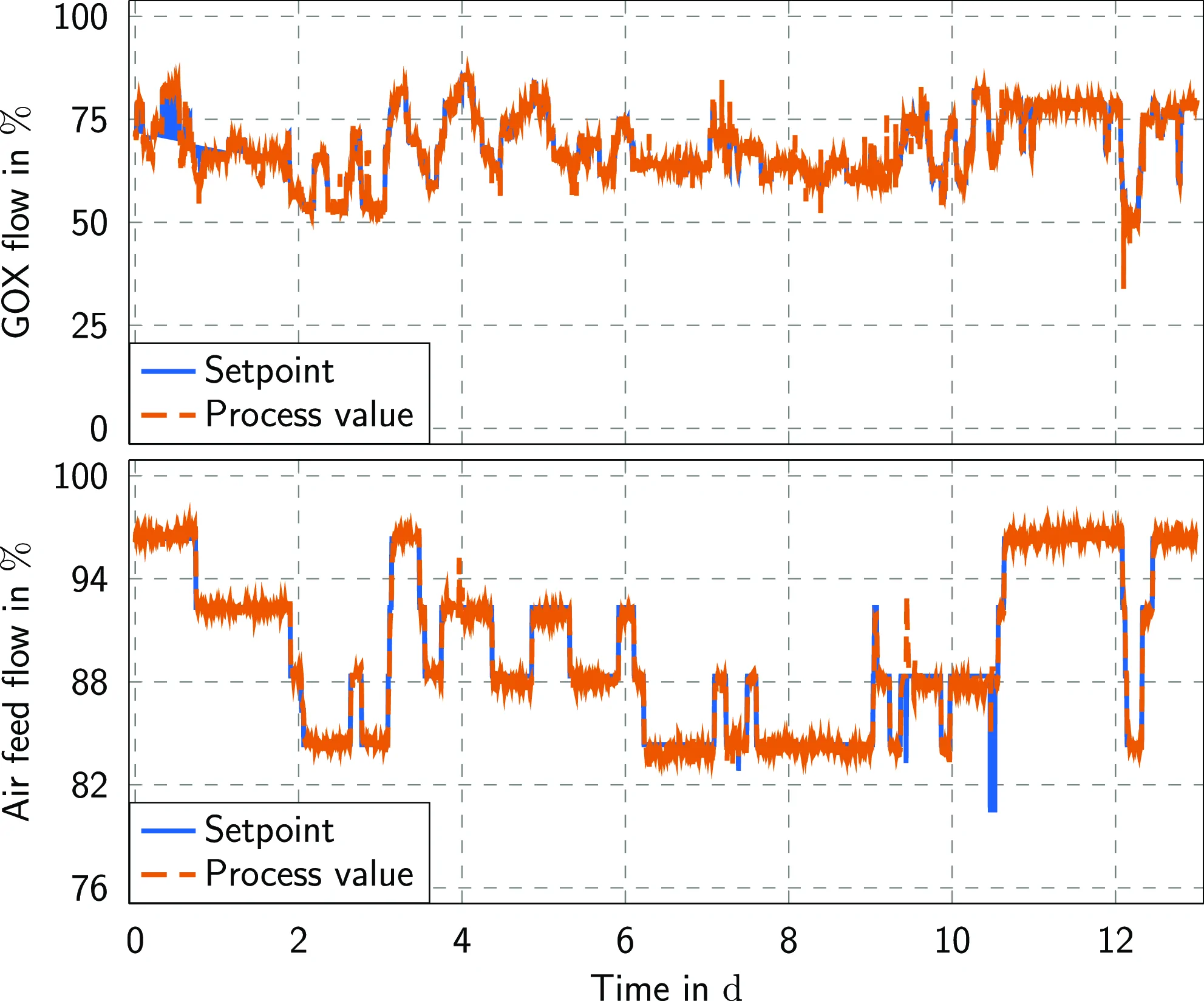
Plant data for GOX and air feed flow.

One of the primary objectives of a production plant is to guarantee the required product specifications. Figure 11 therefore depicts the purity levels for both the GOX and the GAN product. These two measurements can also be seen as indicators of the purity of the LOX and LIN products. This is due to the fact that the gaseous and liquid products of each component are obtained at the same point in the process, thus, the maximum purity difference is the corresponding equilibrium concentration. However, particularly at the present purity levels, this difference is rather small. Therefore, based on a comparison of the data with the product specifications listed in Table 1, this reveals that the required specifications are successfully fulfilled for all products during the entire time period. It is achieved regardless of the current operating mode or operating point of the plant, and regardless of the present control task.

**Figure 11:**
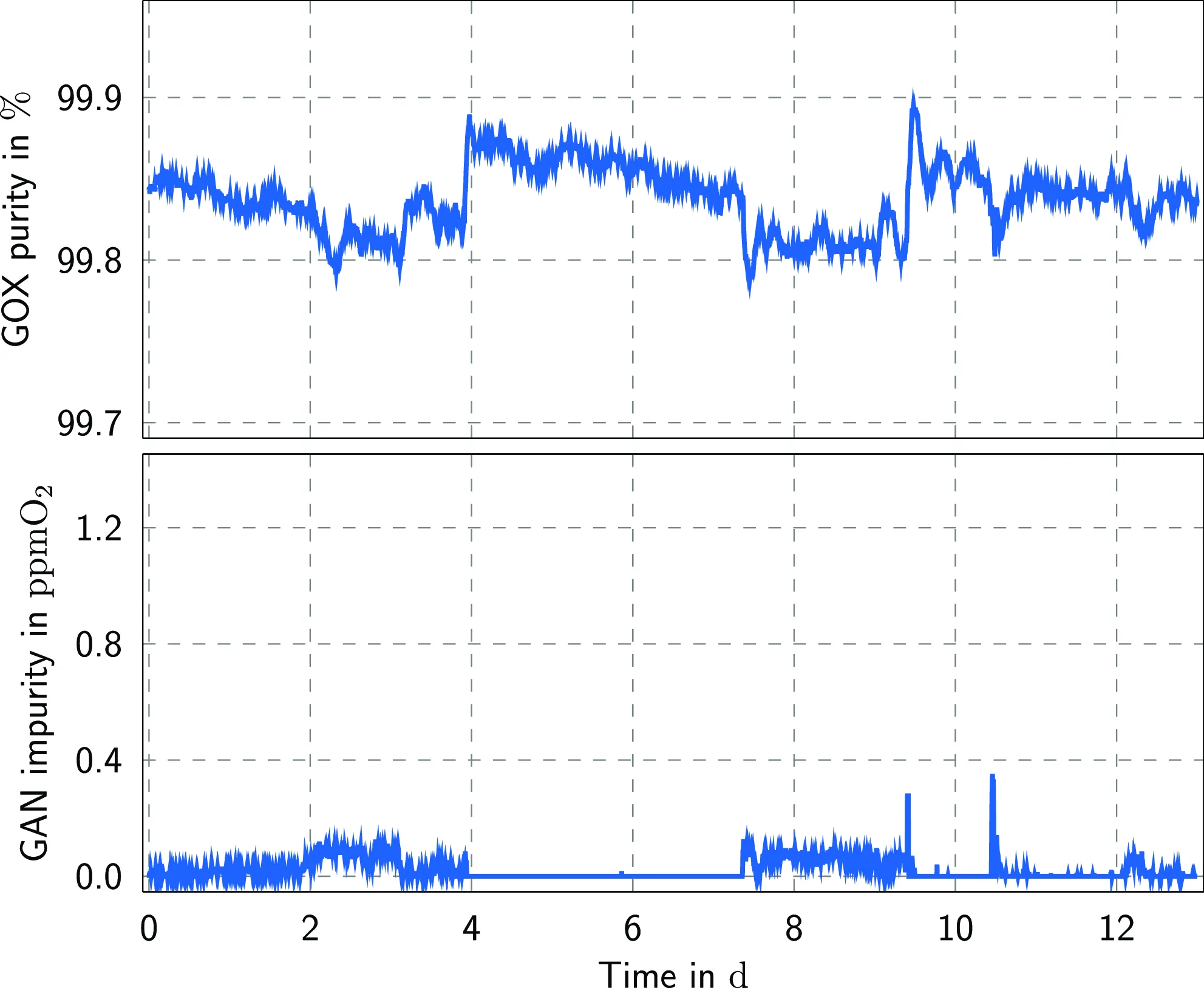
Plant data for purity levels for both the GOX and the GAN product.

The results presented regarding application ASU2 demonstrate AIPC in a real-world application of an in-production-critical ASU. In this context, AIPC is capable of covering different operating modes and control tasks using a single prediction model while meeting the required product specifications. A prerequisite for this is, of course, that the necessary data required for model training is available for both operating modes.

In the following, the performance obtained with AIPC is to be compared with the previously implemented control strategy of the considered plant, which does not use any form of MPC but relies solely on PI controllers and an ALC system. Therefore, Figure 12 shows data from a 20-day period of the considered plant. In the upper plot, the trend of the BAC flow indicates that the plant is operated entirely in liquid mode.

**Figure 12:**
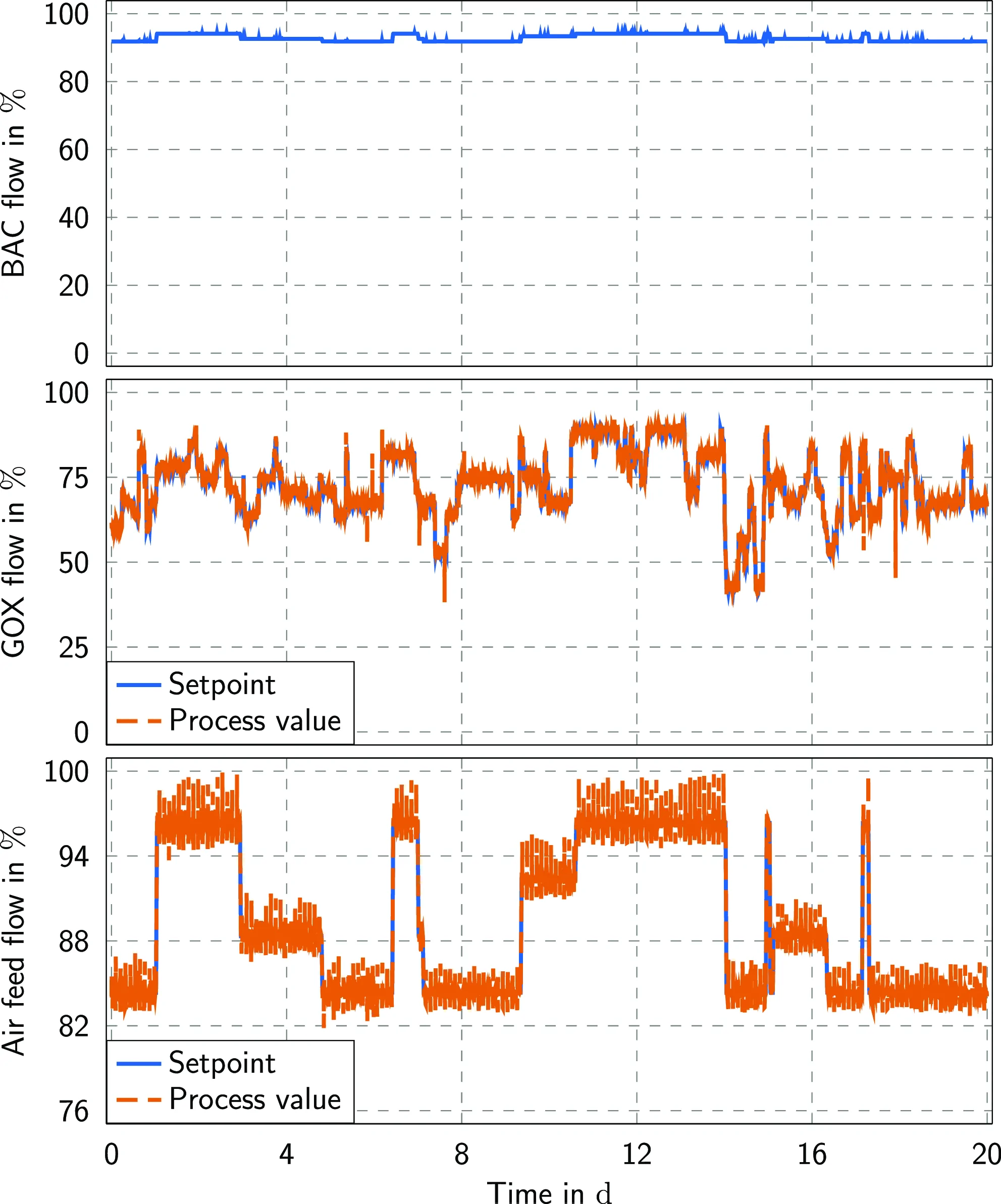
Plant data for the BAC, GOX and air feed flow during the previous control strategie (PI+ALC).

As always, the plant needs to meet changing customer requirements. For this reason, various operating points and load change scenarios were performed, as illustrated by the trends in the GOX product flow and the air feed flow (middle and lower plot of Figure 12). It is worth mentioning that in a real production plant, operation over a longer period of time never completely overlaps with a second period, however, the load changes shown here do correspond to operating behavior similar to that shown in the previous analysis with AIPC control. Therefore, both control strategies can be compared.

A key objective of a production plant is to guarantee the required product specifications. Figure 13 therefore depicts the purity levels for both the GOX and the GAN product.

**Figure 13:**
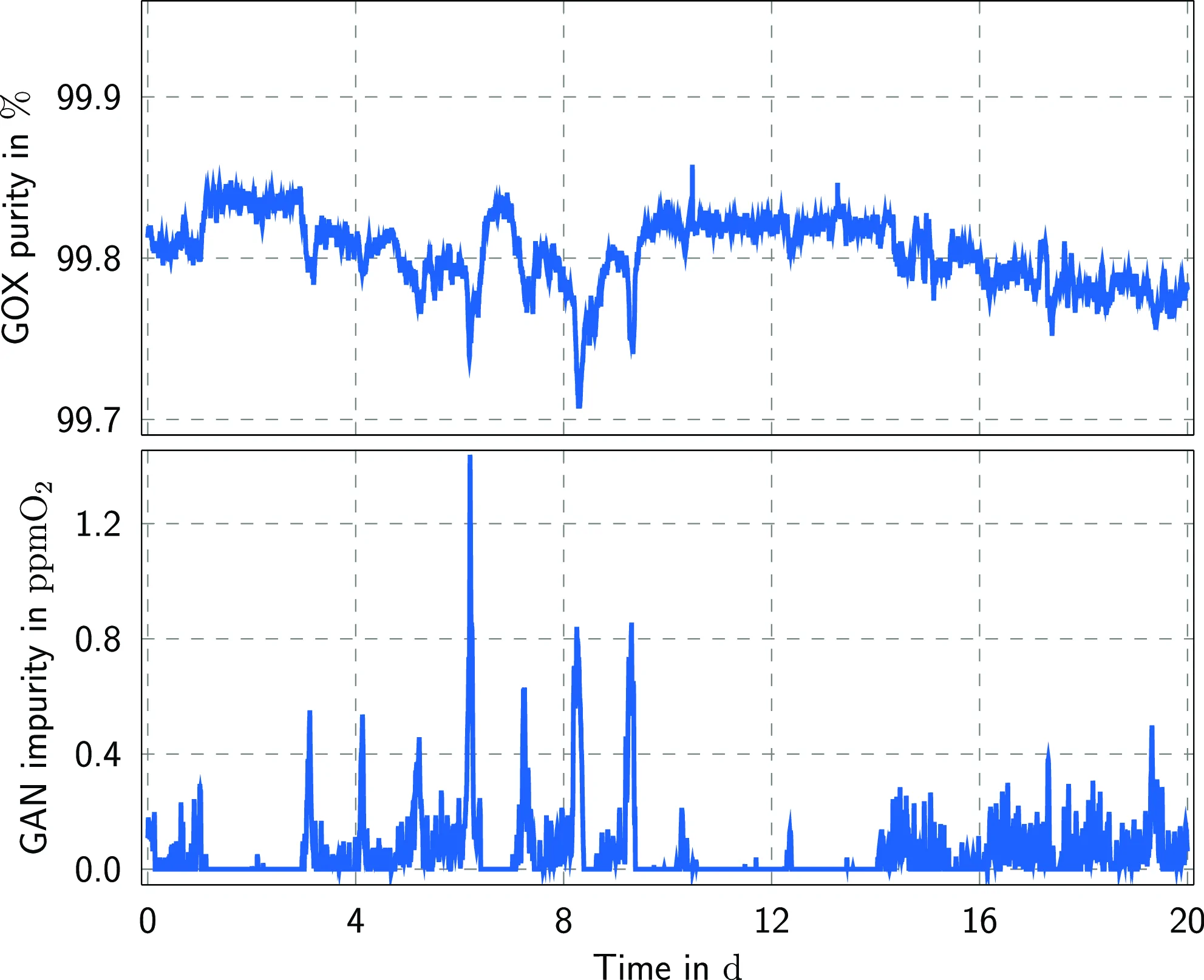
Plant data for purity levels for both the GOX and the GAN product during the previous control strategie (PI+ALC).

The trend of the GOX purity shows that the purity requirement specified in Table 1 is guaranteed throughout the entire time period. However, if this trend is compared with the results shown in Figure 11, which are obtained with active AIPC control, it is clearly evident that AIPC control yields higher purity values. This is also evident when examining the mean values and standard deviations achieved for both control strategies, as shown in Table 5.

**Table 5:** Comparison of Product Specifications for Both Control Strategies (AIPC vs. PI+ALC)

	control strategy	product	mean value	standard deviation
13-day period	AIPC	GOX purity	99.838%	0.019%
		GAN impurity	0.023 ppmO_2_	0.051 ppmO_2_
20-day period	PI+ALC	GOX purity	99.804%	0.021%
		GAN impurity	0.059 ppmO_2_	0.121 ppmO_2_

The same results are observed for the GAN impurity, now to an even greater extent. Consequently, the obtained values are significantly higher compared to the results with active AIPC control, indicating a more impure product. In fact, even a brief violation of the required purity specification is present during the considered time period. Additionally, there are significantly larger fluctuations over time. To be more precise, the mean value in the GAN impurity is now increased by a factor of 2.57 compared to AIPC control, wherease the standard deviation is increased by a factor of 2.37 (see results in Table 5).

The comparison clearly shows that the use of AIPC results in improved control performance compared to the previously installed control strategy, which consisted of PI controllers and an ALC system. Thus, AIPC is not only capable of covering different operating modes and control tasks using a single prediction model but also ensures improved product purities. Furthermore, smaller fluctuations over time can be observed. In general, this allows the plant to be operated more closely to critical product specifications. Although this is not done in the current work for the sake of comparability, it can certainly be implemented in the real plant to ensure more efficient plant operation.

## Conclusion

In summary, a data-driven nonlinear model predictive control framework, referred to as AIPC, is presented in order to control an air separation unit (ASU). This approach, in which the prediction model is an artificial neural network (ANN), requires minimum process knowledge and only relies on historical plant data. In general, AIPC is currently used for control of several in-production-critical *Linde* ASUs.

Since the prediction model is based solely on historical plant data, any nonlinear relationships can be mapped and the entire operating range can be covered, making AIPC a powerful alternative to physically modeled nonlinear model predictive control (NMPC). The approach also enables fast implementation at the plant as step tests are not required, which are usually time-consuming and interfere with the normal operation of the production plant. Occasional retraining of the prediction models also allows the adaptation to changing process conditions. Furthermore, the maintenance effort is reduced compared to classic NMPC solutions.

In the present work, AIPC performance is presented for two different applications. In the first one, multivariable control of a digital twin of an ASU comprising four control loops is considered. Additionally, a multimodel extension is presented that combines several data-driven prediction models in a parallel way. In general, the approach allows to handle control loops with large differences in their settling times, and to group process variables of a particular process area. Consequently, only the main dependencies of these process variables are exploited.

Within the first application, a typical real-world scenario comprising a 20% load change is presented while complying with the required product specifications. Furthermore, it is shown that the presented data-driven MPC approach can even handle a more extreme scenario consisting of a 50% load change while successfully maintaining the required product specifications throughout the entire time period.

In the second application, the performance of AIPC is presented for an in-production-critical *Linde* ASU. Here, two different operating modes including different products, different equipment, and a different overall control task, can be captured by a single nonlinear model without the need for changing the input/output variable sets or control models. It is shown that AIPC achieves the required purity specifications for all products, regardless of the current operating mode or operating point of the plant. Furthermore, AIPC control shows enhanced control performance and improved product purities compared to the previously installed control strategy.

In general, AIPC is process agnostic and can therefore be deployed alongside ASUs for various industrial control applications such as refinery and petrochemical processes. It is a scalable solution with minimal effort on model training by leveraging an automated training framework and represents a nonlinear MPC framework that can be used for the control of industrial plants. Additionally, the multimodel extension helps improving performance and robustness.

## Nomenclature

**Symbols**
c: constraint limit
f: function
J: value of cost function
K: overall number of time steps
k: current time step
L: number of constraints used
M: number of manipulated variables used
N: number of controlled variables used
p: process value of controlled variable
s: set point of controlled variable
u: process value of manipulated variable
**Greek symbols**
α, β, γ: weighting coefficients
**Subscripts**
k: time step
l: constraint
m: manipulated variable
n: controlled variable
**Abbreviations**
AIPC: data-driven nonlinear model predictive control
ALC: automatic load change system
ANN: artificial neural network
ASU: air separation unit
BAC: booster air compressor
CV: controlled variable
DCS: distributed control system
DT: digital twin
GAN: gaseous nitrogen
GAR: gaseous argon
GOX: gaseous oxygen
LAR: liquid argon
LIN: liquid nitrogen
LMPC: linear model predictive control
LOX: liquid oxygen
MPC: model predictive control
MSE: mean squared error
MV: manipulated variable
NARX: nonlinear autoregressive model with exogenous inputs
NMPC: nonlinear model predictive control
PGAN: pressurized gaseous nitrogen
PI: proportional-integral
RNN: recurrent neural network
